# Characterisation of Pelvic Floor Muscle Activation in Adults With Intellectual Disability Using Surface Electromyography: A Comparative Cross‐Sectional Study

**DOI:** 10.1111/jar.70300

**Published:** 2026-08-31

**Authors:** Ana López‐Lezcano, Manuel Rozalén‐Bustín, Sara Cerrolaza‐Tudanca, Mª. Soledad Amor‐Salamanca, Lara Sánchez‐Suarez, Arturo Cano‐Uceda, José Luis Maté‐Muñoz, Pablo García‐Fernández

**Affiliations:** ^1^ Faculty of Health Sciences Alfonso X El Sabio University Madrid Spain; ^2^ Faculty of Nursing, Physiotherapy and Podiatry Complutense University of Madrid Madrid Spain

**Keywords:** intellectual disability, pelvic floor, physical therapy, surface electromyography, urinary incontinence

## Abstract

**Introduction:**

Adults with intellectual disability (ID) have high rates of urinary incontinence, yet objective neuromuscular data are limited. We compared pelvic floor muscle (PFM) activation using surface electromyography (sEMG) in adults with ID versus matched controls.

**Methods:**

In this cross‐sectional study, 104 participants were recruited to an ID group (*n* = 52) and a matched control group (*n* = 52). Perianal sEMG recorded five maximal voluntary contractions following a standardised protocol. Intensity, variability, contraction onset and relaxation times were analysed using *t* or Mann–Whitney tests, MANOVA and multiple regression.

**Results:**

Compared with controls, the ID group showed lower mean activity (5.3 ± 6.0 vs. 8.8 ± 7.2 μV; *p* < 0.001; *d* = 0.52), lower peaks, longer onset and greater variability; relaxation parameters did not differ. MANOVA indicated a significant global group effect (Wilks' *λ* = 0.801, *p* = 0.003). Regression identified ID as the sole independent predictor of reduced activation.

**Conclusions:**

Adults with ID exhibit weaker, less stable and delayed PFM activation, supporting tailored physiotherapy.

## Summary

- We compared pelvic floor muscle activation in 104 adults, 52 with intellectual disability and 52 without, using surface electromyography measured with adhesive skin sensors.
- Adults with intellectual disability showed contractions that were weaker, less stable and slower to start; relaxation did not differ substantially between groups.
- These findings support simple, adapted pelvic‐floor training, as well as the incorporation of routine non‐invasive assessments to establish baseline values, individualise therapy and monitor progress.
- We provide a reproducible protocol and reference values for assessing pelvic‐floor function in adults with intellectual disability, laying the groundwork for intervention trials and longitudinal studies.

## 1 Introduction

Intellectual disability (ID) is the most prevalent neurodevelopmental disorder, characterised by significant limitations in intellectual and adaptive functioning, with a sustained impact throughout the individual's life and on their family and social environment. Its prevalence is estimated at around 3% of the general population, and its aetiology continues to be the subject of active research given its clinical and genetic heterogeneity (Benmakhlouf et al. 2021).

ID is associated with a higher incidence of urological and gastrointestinal problems, notably nocturnal enuresis, daytime urinary incontinence (UI), faecal incontinence and constipation (von Gontard et al. 2022). It is estimated that between 26% and 52% of adults with ID have UI, which represents a significant factor in functional impairment and quality of life (Finlayson, Gore, et al. 2025). In addition, adults with ID have been found to have an increased prevalence of chronic conditions such as constipation, UI, thyroid disorders and obesity compared to older adults without ID (García‐Domínguez et al. 2020).

From a functional point of view, the pelvic floor (PF) consists of a complex network of muscles, ligaments and fascia that provide structural support to key organs such as the bladder, rectum and reproductive organs. Anatomically, there are superficial muscles, such as the bulbospongiosus, ischiocavernosus and transverse perineal muscles and deep muscles such as the levator ani (formed by the puborectalis, pubococcygeus and iliococcygeus) and coccygeus. Innervation is provided by the pudendal nerve and sacral roots S3–S5, which are essential for sensory, motor and continence function (Eickmeyer 2017).

The pelvic floor muscle (PFM) actively participates in continence control through coordinated reflex and voluntary contractions, especially in response to increases in intra‐abdominal pressure (Eickmeyer 2017). UI, defined as the involuntary loss of urine, can be classified into different clinical subtypes, including stress, urge, mixed, overflow or functional and is often associated with alterations in the neuromuscular control of the bladder and/or the PF muscles (Irwin 2019). Among the modifiable risk factors, PF muscle weakness is one of the most relevant (Blomquist et al. 2020).

Objective assessment of PF muscle function is key to both clinical evaluation and the design of personalised training programmes. In this regard, electromyography (EMG) allows the recording of the electrical potential generated by the motor unit during muscle contraction and can be performed using surface or invasive electrodes. Its application has been widely validated to quantify contraction intensity, coordination, fatigue and motor recruitment patterns (Eickmeyer 2017; Bø and Sherburn 2005; American Association of Neuromuscular and Electrodiagnostic Medicine (AANEM) 2011).

In the urological field, EMG of the anal or urethral sphincter is a useful tool in urodynamic assessment and in the diagnosis of neurogenic and non‐neurogenic PF disorders, using devices that may include needle electrodes, perianal surface electrodes, anal plugs or multi‐electrode systems (Garcia and Vieira 2011).

UI in adults with ID is considered a multifactorial condition, with functional and mixed UI being among the most common forms (Finlayson, Skelton, et al. 2025; Williams et al. 2024). In addition to possible lower urinary tract dysfunction, cognitive, behavioural and motor factors, as well as difficulties in toileting access, may contribute to continence problems in this population (National Institute for Health and Care Excellence 2019). Furthermore, continence depends on adequate neuromuscular coordination and PFM activation and previous studies suggest that alterations in motor control and neuromuscular efficiency may influence some individuals with ID (Borji et al. 2014). Therefore, UI in this population should not be understood as arising from a completely different aetiology compared to the general population, but rather as the result of a more complex interaction between neuromuscular, cognitive and behavioural factors. Consequently, although standard assessment and treatment protocols may still be applicable, adaptations in communication and motor learning strategies are likely necessary to ensure their effectiveness in adults with ID (National Institute for Health and Care Excellence 2019).

Despite advances in assessment techniques, there is a knowledge gap regarding the electromyographic behaviour of the PFM in people with intellectual disabilities. The current literature does not include studies that objectively and quantitatively analyse the neuromuscular function of the PF in this population, which prevents understanding of possible underlying physiological alterations, hinders the design of specific interventions and perpetuates therapeutic care based on intuition rather than evidence. It is therefore essential to characterise the activation of the PFMs in people with intellectual disabilities using validated tools such as surface electromyography (sEMG), in order to lay the foundations for a physiotherapy approach based on objective data. Therefore, the objective of this study was to characterise, using sEMG, the activation and relaxation of the PF in adults with intellectual disabilities compared to controls without intellectual disabilities, matched for sex and age, evaluating parameters of intensity, variability and muscle response times.

## 2 Methodology

An observational, cross‐sectional, comparative study was conducted to analyse differences in PFM activation between adults with and without intellectual disabilities, using sEMG. This study was approved by the Ethics Committee of Alfonso X el Sabio University (Madrid, Spain) Code 2025_02/349, was conducted in accordance with the ethical principles set out in the Declaration of Helsinki (World Medical Association 2025) and followed the guidelines of the STROBE (Strengthening the Reporting of Observational Studies in Epidemiology) declaration for observational studies (von Elm et al. 2007). All participants, or their legal guardians when necessary, signed the informed consent form before starting the assessment.

Participants were recruited through convenience sampling at two centres: Fundación Los Pueyos (Villamayor, Zaragoza, Spain), dedicated to the care of individuals with intellectual disabilities and the Administrative and Service Staff of Universidad Alfonso X el Sabio (Villanueva de la Cañada, Madrid, Spain). Recruitment was conducted in June 2025 and data collection took place between July and August of the same year.

Inclusion criteria required participants to be between 18 and 65 years of age, able to understand and follow simple instructions and able to participate voluntarily in the assessment procedure. For participants with ID, informed consent was obtained from their legal guardians in accordance with ethical requirements, while participant assent was sought whenever possible. In the ID group, all participants (*n* = 52; 100%) required consent from their legal guardians. Participants in the control group provided informed consent independently. Regarding the level of ID, the sample included individuals with mild (*n* = 29) and moderate (*n* = 23) ID, based on clinical records. Participants were excluded if they had current UI, a previous diagnosis of PF dysfunction, active oncological disease, history of pelvic surgery, pregnancy or recent postpartum, current use of medication with potential neuromuscular effects or motor, sensory or behavioural limitations that could interfere with proper test performance.

A total of 104 participants were included and recruited into two groups: an experimental group (*n* = 52) consisting of individuals with ID and a control group (*n* = 52) composed of adults without ID, matched by age and sex to reduce selection bias. At baseline, demographic (age, sex) and anthropometric data were collected.

PF neuromuscular function was assessed using sEMG with the NeuroTrac MyoPlus 4 Pro device (Verity Medical Ltd., United Kingdom). This device has been previously validated and allows reliable recording of electromyographic activity in different muscle groups, including the PFMs (Singh and Reddy 2018; Oliveira, Silva, et al. 2022; Oliveira, Dantas, et al. 2022; Hennemann and Bayón 2021; Lakens 2022; Costilla et al. 2020; Torres‐Vega et al. 2023). In the present study, only the EMG channels were used for data acquisition. Additionally, electromyographic biofeedback was used during the familiarisation phase to provide real‐time visual feedback on muscle activation and facilitate task learning.

**FIGURE 1:**
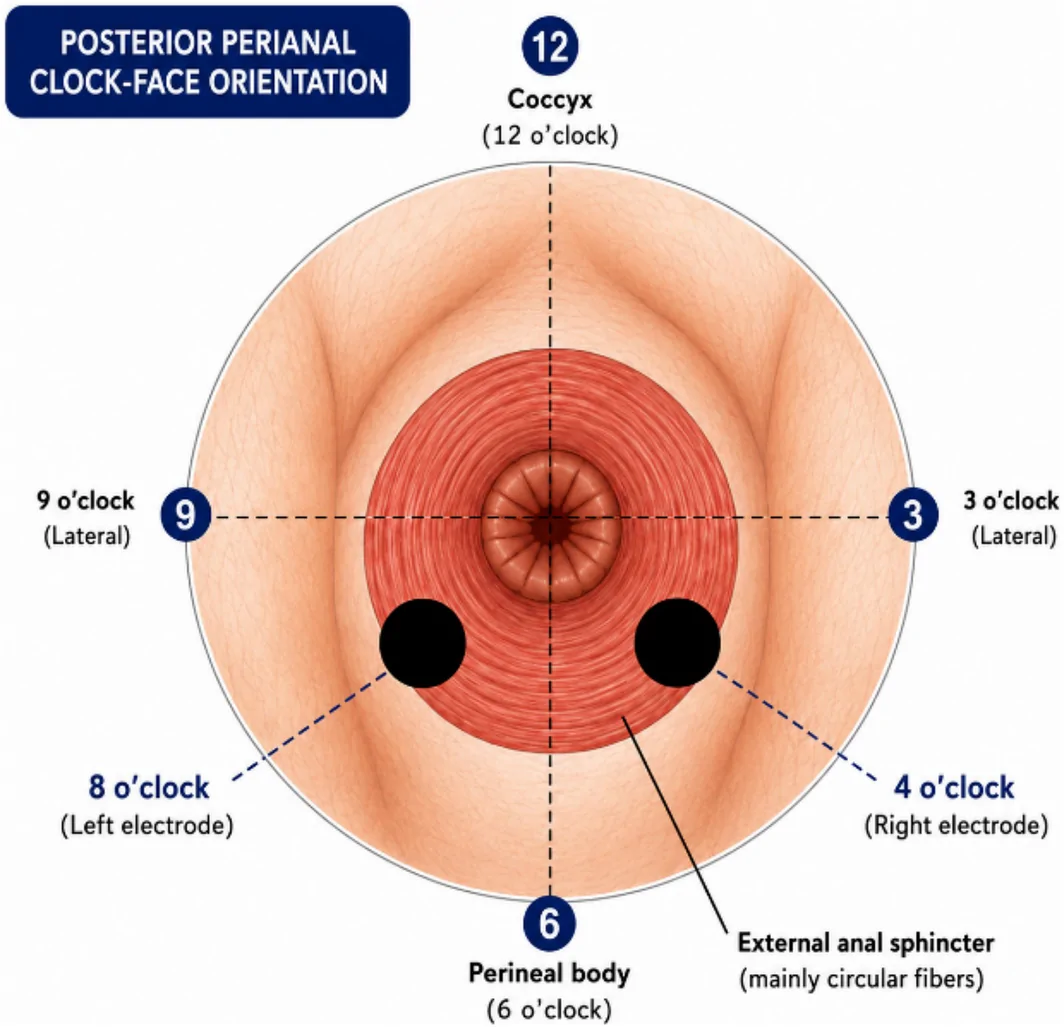
Schematic representation of sEMG electrode placement using a posterior perianal clock‐face orientation. *Note:* The coccyx corresponds to the 12 o'clock position and the perineal body to the 6 o'clock position. Recording electrodes were placed at the 4 and 8 o'clock positions over the external anal sphincter.

A bipolar sEMG configuration was used. As shown in Figure 1, in a posterior perianal clock‐face orientation, the coccyx was designated as the 12 o'clock position and the perineal body as the 6 o'clock position. Two self‐adhesive surface recording electrodes were placed with their active conductive areas cantered approximately at the 4 and 8 o'clock positions, directly over the external anal sphincter. The inter‐electrode distance was 20 mm centre‐to‐centre and the conductive surfaces were kept separated to avoid contact at the midline. A reference electrode was placed over the right anterior superior iliac spine. The electrodes used were NeuroTrac (Verity Medical Ltd., United Kingdom), model NeuroTrac Disposable Surface Electrodes, incorporating self‐adhesive conductive hydrogel. Although these electrodes are supplied as 30‐mm disposable surface electrodes, the active conductive recording area was carefully trimmed to approximately 15 mm before placement, using the same standardised procedure in all participants. This adaptation was performed to ensure that the active conductive surface remained confined within the margins of the external anal sphincter muscle belly and to prevent overlap or contact between the two recording electrodes at the midline.

This electrode placement was intended to record electromyographic activity predominantly from the external anal sphincter region, as part of the PF striated muscle complex, while minimising interference from adjacent muscle groups and avoiding invasive procedures, which is particularly relevant in vulnerable populations such as individuals with intellectual disabilities.

No normalisation procedure was applied in this study; therefore, recorded values were expressed in absolute microvolts (μV). This decision was made to facilitate cooperation among individuals with ID and reduce task burden. To minimise inter‐subject and inter‐recording variability and ensure data reliability, evaluation conditions were rigorously standardised: 15 mm circular surface electrodes were always placed in the same anatomical location after skin preparation; the same device (NeuroTrac MyoPlus 4 Pro) with identical recording settings was used for all measurements; and assessments were systematically conducted by the same physiotherapist experienced in EMG, thereby reducing inter‐rater error and selection bias.

Pre‐test instructions were provided verbally to all participants. In individuals with ID, these instructions were adapted using simple and clear language and when necessary, support from caregivers or familiar staff was provided. Understanding of the instructions was verified during the familiarisation phase prior to data collection, ensuring that participants were able to follow the procedure correctly. Although structured visual materials were not systematically used, communication was adapted to each participant's level of comprehension.

Before formal recording, all participants underwent a familiarisation phase in which they were instructed to perform PF contractions using simple verbal cues (e.g., ‘contract as if trying to stop passing gas or interrupt the flow of urine’). In participants with ID, instructions were adapted to their level of comprehension using simplified language, repetition and immediate verbal feedback. Additionally, electromyographic biofeedback provided real‐time visual information about muscle activation, facilitating motor learning. The formal evaluation protocol consisted of five maximal voluntary PF contractions, each lasting 5 s and separated by 10‐s rest periods to minimise fatigue and allow recovery of the electromyographic baseline. For analysis, the four most consistent recorded contractions were retained according to predefined quality criteria and the representative value for each EMG parameter was derived from these contractions. Artefact exclusion criteria included peaks > 3 SD above the mean contraction value, signal saturation or discontinuity. All tests were performed in the supine position with knees flexed, under standardised verbal instructions. This procedure was based on the protocol described by Castro‐Pardiñas et al. (Castro‐Pardiñas et al. 2016), adapted to the characteristics of the present study and population.

External factors that could affect EMG signals were also controlled: participants were instructed to attend sessions with an empty bladder, to avoid intense physical exercise and the consumption of caffeine/stimulants during the 24 h preceding the test and all evaluations were scheduled in the morning to reduce variability related to fatigue or circadian rhythm.

Electromyographic signals were recorded using the portable NeuroTrac MyoPlus 4 Pro system (Verity Medical Ltd., United Kingdom). The device's ‘Wide’ band‐pass filter (≈18–370 Hz) and 50 Hz notch filter were applied to reduce motion artefacts and power line interference. The system provided electromyographic amplitude values in microvolts (μV) through internal rectification and RMS algorithms. For each maximal voluntary contraction of the PF, the following parameters were quantified. The primary outcome was the mean activity (μV) during contraction. Secondary outcomes included resting activity (μV), standard deviation (SD) during contraction and rest, maximum RMS value (μV), post‐contraction signal reduction (μV) and onset and relaxation times (s). Contraction onset was defined as the first point where the RMS signal exceeded baseline by more than 3 SD for at least 150 ms, while relaxation was defined when the signal returned below this threshold.

The choice of this technique reflects the need for an objective, non‐invasive and validated measure capable of detecting potential functional differences between individuals with and without ID, thereby contributing to the identification of neuromuscular activation patterns that may inform future interventions in the field of urogynecological physiotherapy.

### 2.1 Calculation of Sample Size

Before data collection, we performed an a priori sample size calculation in G*Power (v3.1.9.7) for a two‐tailed independent‐samples comparison (*α* = 0.05; 1 − *β* = 0.80). In the absence of prior studies in this specific population, we grounded the calculation in a smallest effect size of interest (SESOI) rather than in uncertain estimates from other populations. We prespecified a moderate standardised effect (Cohen's *d* = 0.50) as clinically meaningful and plausible for between‐group differences in the primary outcome. Under these assumptions, the required minimum was *n* = 45 per group (*N* = 90). To accommodate matching and potential attrition, we planned *n* = 52 per group (*N* = 104), which was achieved. This a priori calculation set our minimum recruitment target and is reported for transparency; statistical inference relies on effect sizes and 95% CIs (not on post hoc power). The use of SESOI‐based planning and equivalence bound logic follows current methodological recommendations (Lakens 2022).

### 2.2 Statistical Analysis

Statistical analyses were performed using IBM SPSS Statistics v29.0 (IBM Corp., Armonk, NY, USA). First, a descriptive analysis of the included variables was carried out. Quantitative variables were summarised as mean and SD, while qualitative variables were expressed as absolute frequencies and percentages. The normality of quantitative variables and the homogeneity of variances were assessed before applying the corresponding parametric tests.

For comparison of the primary variable, PFM activation measured by sEMG, between the two study groups, an independent‐samples Student's *t*‐test was used when assumptions of normality and homogeneity were met. In case of assumption violations, the non‐parametric Mann–Whitney *U* test was applied. The effect size for Student's *t*‐test was determined using Cohen's *d*, with thresholds defined as small (0.20–0.49), medium (0.50–0.79) and large (≥ 0.80) (Cohen 1973). The effect size for the Mann–Whitney *U* test was calculated using the formula *r* = *Z*/√*n* (Tomczak and Tomczak 2014).

To evaluate the overall effect of ID on the set of electromyographic variables related to PF activation, a multivariate analysis of variance (MANOVA) was conducted, using group membership (ID vs. control) as the independent variable. Wilks' lambda and Pillai's trace statistics were used to assess model significance, along with their associated *F* values, degrees of freedom, *p* values, partial *η* ^2^ and observed power.

Finally, to control for potential confounding variables and obtain an adjusted analysis, a multiple linear regression model was developed, with PF activation measured by EMG as the dependent variable and group membership (with vs. without ID), sex, age and anthropometric variables as independent predictors. Multicollinearity among predictors was examined and model fit was evaluated using the adjusted coefficient of determination (adjusted *R* ^2^). A *p* value < 0.05 was considered statistically significant for all analyses.

To minimise risk of bias, the data analyst remained blinded to group allocation. Records were anonymised with coded identifiers and unblinding occurred only after obtaining the final results.

## 3 Results

A total of 104 participants were included, with 52 individuals in each group. No significant differences were observed between the groups in terms of age, gender distribution, weight or body mass index (BMI). However, the control group was significantly taller than the group with disabilities (Table 1).

**TABLE 1:** Descriptive statistics of the included participants.

Variable	Intellectual disability group (*n* = 52)	Control group (*n* = 52)	*p*
Age (years)	42.3 ± 6.2	42.7 ± 4.6	NA, matched variable
Gender			
Male (*n*, %)	28 (54)	28 (54)	NA
Female (*n*, %)	24 (46)	24 (46%)	
Weight (kg)	65.4 ± 14.0	67.2 ± 13.9	0.421
Height (cm)	162.6 ± 13.1	167.0 ± 7.9	0.043
BMI (kg/m^2^)	19.4 ± 3.4	20.0 ± 3.5	0.810

As shown in Table 2, the analysis of the electromyographic variables showed significant differences between the groups in several of the parameters evaluated. The control group had higher mean activity values during PF contraction, as well as a lower standard deviation of the signal during this contraction. Likewise, the mean time to the onset of contraction was lower in the control group. Significant differences were also observed in the maximum value recorded during voluntary contraction, with higher figures in the control group. However, no statistically significant differences were found between the groups in the mean reduction of the signal after contraction of activity, in the variability of the signal during rest or in the mean relaxation time after contraction.

**TABLE 2:** Comparison of pelvic floor electromyographic parameters between groups.

		Intellectual disability group (*n* = 52)	Control group (*n* = 52)	*p*	Effect size
Average activity (μV)	Mean ± SD	5.3 ± 6.0	8.8 ± 7.2	0.001	0.52
	(95% CI)	(3.6–7.0)	(6.8–10.8)		
Average signal reduction after contraction (μV)	Mean ± SD	2.6 ± 3.1	2.4 ± 4.4	0.979	0.04
	(95% CI)	(1.7–3.5)	(1.2–3.7)		
Deviation activity (μV)	Mean ± SD	1.5 ± 1.3	0.9 ± 1.1	0.001	0.47
	(95% CI)	(1.1–1.9)	(0.6–1.2)		
Deviation rest (μV)	Mean ± SD	0.5 ± 0.6	0.4 ± 0.5	0.405	0.22
	(95% CI)	(0.3–0.7)	(0.2–0.5)		
Maximum (μV)	Mean ± SD	17.8 ± 21.6	19.0 ± 12.8	0.002	0.26
	(95% CI)	(11.8–23.9)	(15.4–22.5)		
Average start (s)	Mean ± SD	0.8 ± 0.2	0.6 ± 0.4	< 0.001	0.67
	(95% CI)	(0.8–0.9)	(0.5–0.7)		
Average relaxation (s)	Mean ± SD	0.5 ± 0.2	0.5 ± 0.2	0.627	0.14
	(95% CI)	(0.4–0.6)	(0.4–0.5)		

**FIGURE 2:**
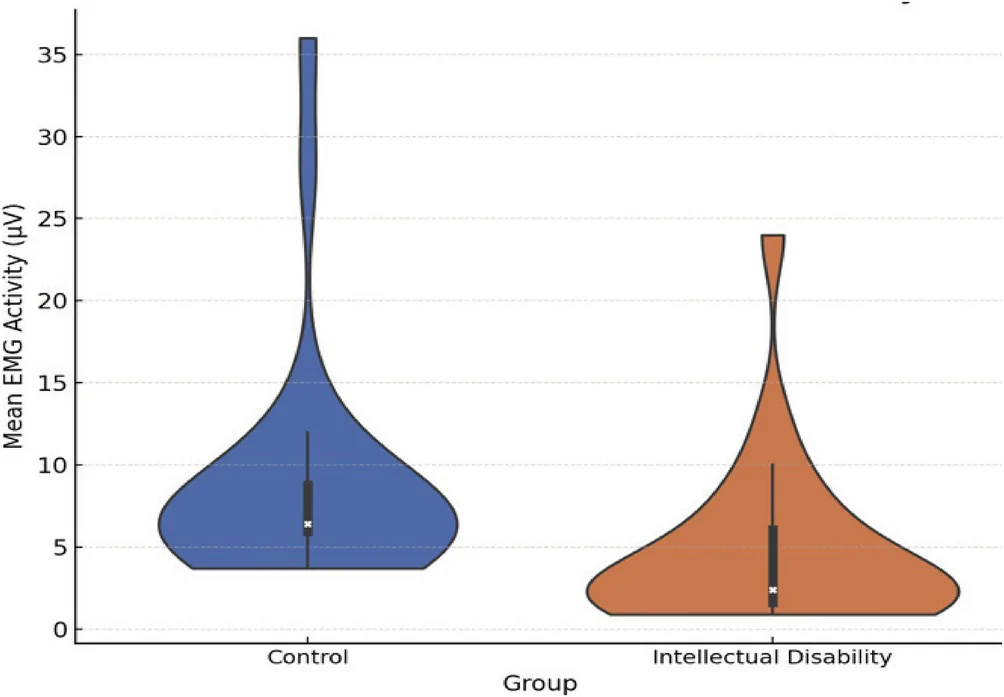
Distribution of mean pelvic floor EMG activity in adults with and without intellectual disability.

In Figure 2, the distribution of mean PF activity measured by EMG is presented for both groups. Participants with ID showed significantly lower values and greater interindividual variability, whereas the control group exhibited higher values and a more compact distribution. This graphical representation complements the data shown in Table 2, highlighting both the reduced activation capacity and the less stable recruitment pattern observed in the disability group.

As shown in Table 3, the multivariate linear model showed a significant effect of the group on the set of PF electromyographic variables. Specifically, individuals with ID showed a significantly lower activation pattern compared to controls, reflecting lower intensity and efficiency in the PF neuromuscular response. This effect was associated with a considerable proportion of the variance explained by the model, reinforcing its clinical and functional relevance. Likewise, the high statistical power achieved in the model supports the robustness of the results and reduces the probability of Type II error, confirming that the sample size was sufficient to detect real differences between the groups.

**TABLE 3:** Multivariate linear model for the effect of group (with intellectual disability vs. without intellectual disability) on the set of pelvic floor electromyographic variables.

	Value	*F*	Hypothesis df	Error df	Sig.	Partial *η* ^2^	Observed power
Pillai's trace	0.199	3.409	7.000	96,000	0.003	0.199	0.953
Wilks' lambda	0.801	3.409	7.000	96,000	0.003	0.199	0.953

As shown in Table 4, the multiple linear regression analysis showed that ID was the only predictor significantly associated with lower mean PF activation, once adjusted for age, sex and BMI. Neither age, sex nor BMI showed significant associations with the dependent variable.

**TABLE 4:** Multiple linear regression model for pelvic floor activation (mean activity in μV).

Model	Unstandardised coefficients		Standardised coefficients	*t*	Sig.	Collinearity statistics	
	*B*	SE	*β*			Tolerance	VIF
Constant	7.521	4.612		1.631	0.106		
Disability	−3.507	1.305	−0.257	−2.688	0.008	0.999	1.001
Age	0.096	0.064	0.146	1.498	0.137	0.960	1.041
Gender	−0.969	1.319	−0.071	−0.735	0.464	0.978	1.022
Bmi	−0.112	0.195	−0.056	−0.577	0.565	0.964	1.037

It should be noted that the variables of height and weight were excluded from the final model due to the collinearity detected with BMI, which was selected as the representative anthropometric indicator. In addition, collinearity statistics (tolerance and VIF) indicated adequate independence between the included predictors, supporting the robustness of the model.

## 4 Discussion

The main objective of this study was to compare PF electromyographic activity between individuals with intellectual disabilities and a control group without disabilities, matched for sex and age. To our knowledge, this is the first study to specifically and systematically analyse the neuromuscular activation characteristics of the PF in individuals with intellectual disabilities, using quantitative measures obtained through sEMG. The scarcity of previous research focusing on this group hinders both the understanding of the possible mechanisms involved in PF dysfunction and the development of therapeutic strategies adapted to their needs. Therefore, the findings of this study provide novel and relevant information on the functional differences that may underlie the high prevalence of UI described in this population (Finlayson, Skelton, et al. 2025). Furthermore, the presence of motor, sensory and cognitive dysfunction in this group described in the literature (van Schrojenstein Lantman‐De Valk et al. 2000) could reflect the existence of specific alterations in the PF activation pattern that have not yet been characterised.

In terms of the anthropometric characteristics of the sample, both groups were matched for age and sex distribution, which reinforces the validity of the comparisons made. No significant differences were observed in body weight or BMI; however, the group with ID was significantly shorter than the control group. This difference, although modest, could reflect phenotypic characteristics associated with certain genetic syndromes present in this population, as previously described in the literature (Bertapelli et al. 2014; Lima et al. 2024; Pornprasertchai et al. 2020). However, since the effect of anthropometric variables was controlled for in the multivariate models, it is unlikely that this difference had a substantial influence on the main results of the study.

Comparative analysis of electromyographic variables revealed significant differences in several key parameters of PF activation. First, the average activity recorded was significantly lower in the group with ID, suggesting a lower capacity for voluntary muscle recruitment in this population. In addition, greater variability was observed during contraction (activity deviation), which could reflect a less efficient or more unstable activation pattern that has been described in people with intellectual disabilities (Vuijk et al. 2010). Likewise, the group with disabilities showed a slower onset of contraction after the verbal command to contract, which may indicate alterations in neuromuscular coordination or voluntary motor response capacity. Similarly, the maximum signal also showed significantly lower values in the group of people with ID. The deviation at rest was also slightly lower in the group with ID, although these differences did not reach statistical significance. The absence of differences in the relaxation phase suggests that the main deficit is concentrated in the active phase of contraction, rather than in recovery. Taken together, these findings reinforce the hypothesis that individuals with ID have an altered muscle activation pattern, which also affects the PF and which could contribute to the high prevalence of UI described in this population (Finlayson, Skelton, et al. 2025; Bossink‐Tuna et al. 2009; Hsu et al. 2006; Madill and McLean 2008).

The results observed in the population with ID show similarities with the patterns described in subjects with PF dysfunction, such as stress UI, chronic pelvic pain or overactive bladder. In both conditions, lower contraction intensity and reduced electromyographic signal stability have been described, reflecting less efficient neuromuscular recruitment (Ptaszkowski et al. 2020; Bennink 2020). However, while in PF dysfunction these findings are usually related to peripheral alterations in the musculature and supporting tissue, in the case of ID they could be linked to more global deficits in motor coordination and central neuromuscular control. This convergence in results suggests that physiotherapy and PF training programmes used in the general population with dysfunction could serve as a reference for people with ID, although their design would need to be adapted to the cognitive and functional characteristics of these individuals.

The results of the multivariate linear model confirmed the existence of a significant overall effect of the group on the set of electromyographic variables analysed. This finding suggests that, beyond specific differences in individual variables, there is a generalised pattern of altered muscle activation in the group with ID. These data reinforce the idea that PF dysfunction in this population cannot be attributed to a single parameter but rather responds to a broader alteration in neuromuscular control, possibly associated with cognitive, sensory or motor deficits characteristic of people with ID (Absoud et al. 2022; Sadozai et al. 2024; Williams et al. 2022; Lewin et al. 2025; Pan et al. 2017; Pienaar et al. 2015).

The multiple linear regression model allowed us to identify which variables significantly explained the variability in the mean electromyographic activity of the PF. Of all the variables included, only the presence of ID showed a statistically significant association with lower muscle activation, even after adjusting for age, sex and BMI. This finding reinforces the relevance of ID as an independent factor associated with a decrease in the neuromuscular response of the PF. Neither age nor sex showed significant associations in this model, suggesting that, in this matched sample, these factors do not explain substantial differences in the recorded activity. Although BMI was included in the multivariable model because of its potential influence on PF function, BMI values were relatively low and comparable between groups and no significant association with PFM activation was observed. Therefore, body composition is unlikely to have meaningfully contributed to the differences identified between groups in the present study. Taken together, these results confirm that ID is a key determinant in the alteration of the PF activation pattern, beyond other individual characteristics.

The findings of the present study should be interpreted within the multifactorial nature of urinary continence in adults with ID. Although PF dysfunction cannot be considered the sole mechanism involved, the altered muscle activation patterns observed may reflect differences in motor control and neuromuscular coordination. Our results do not suggest a completely different aetiology from that of the general population, but rather a more complex interaction between neuromuscular, cognitive and behavioural factors that may influence toileting behaviours, treatment adherence and the execution of voluntary contractions (Finlayson, Skelton, et al. 2025; National Institute for Health and Care Excellence 2019). In this context, standard PF assessment and rehabilitation protocols may still be useful, although they likely require methodological and communication adaptations to meet the specific support needs of adults with ID (National Institute for Health and Care Excellence 2019).

The present study has some limitations that should be acknowledged. First, the use of sEMG does not allow for precise differentiation between the different PFMs, nor does it ensure absolute quantification of the force generated, which could limit the generalisation of the results. Furthermore, although the groups were matched for gender and age, it was not possible to control for other potentially influential variables, such as the degree of disability, the presence of neurological comorbidities or physical training history. In addition, the sample included individuals with different levels of comprehension and collaboration, which may have affected the performance of motor tasks, especially in the group with disabilities. Finally, only voluntary PFM contractions were assessed. Although voluntary activation has direct clinical relevance in PF rehabilitation, the assessment of reflex contractions, particularly those elicited during coughing, also represents a standard component of clinical PF evaluation and provides valuable information regarding automatic neuromuscular control. Reflex contractions were not included in the present study because ensuring their standardised and reliable execution was considered particularly challenging in adults with ID due to variability in comprehension, collaboration and motor response consistency. Consequently, the present findings do not provide information regarding reflex PF function.

The results obtained have important clinical implications. The lower activation and greater instability in PF contraction observed in the group with ID could contribute to the high prevalence of UI documented in this population. Therefore, these findings support the need to develop specific intervention programmes tailored to the cognitive and functional characteristics of people with intellectual disabilities. Interventions based on PF physiotherapy, motor control training and health education could be particularly beneficial if designed with an accessible, motivational and progressive approach.

Future studies should further investigate the pathophysiological mechanisms underlying PF dysfunction in adults with ID, including the assessment of reflex PF responses, particularly cough‐induced contractions, to obtain a more comprehensive characterisation of PF function. Additional research should also explore the relationship between PFM activation patterns, UI subtypes and functional or cognitive variables, as well as the effectiveness of adapted and personalised PF rehabilitation programmes and their impact on continence, quality of life and personal autonomy.

## 5 Conclusions

The results of this study show that individuals with intellectual disabilities have significantly lower and more unstable electromyographic activation of the PF compared to a control sample matched for sex and age. These neuromuscular alterations could contribute to the high prevalence of UI in this population. Multivariate analysis confirmed an overall pattern of altered activation and multivariate regression identified ID as an independent factor associated with lower muscle activation. Taken together, these findings reinforce the need to design specific interventions tailored to their functional characteristics and to promote further research to improve their urogynaecological health and quality of life.

## Author Contributions

**Ana López‐Lezcano:** conceptualisation, methodology, formal analysis, investigation, resources, writing – original draft, project administration. **Manuel Rozalén‐Bustín:** conceptualisation, methodology, formal analysis, investigation, data curation, resources, writing – original draft, project administration. **Sara Cerrolaza‐Tudanca:** investigation, data curation, visualisation, writing – review and editing. **Mª Soledad Amor‐Salamanca:** investigation, data curation, writing – review and editing. **Lara Sánchez‐Suarez:** investigation, resources. **Arturo Cano‐Uceda:** investigation, data curation, visualisation, writing – review and editing. **José Luis Maté‐Muñoz:** investigation, validation, resources, supervision, writing – review and editing. **Pablo García‐Fernández:** methodology, formal analysis, investigation, data curation, validation, resources, supervision, writing – original draft, project administration.

## Funding

The authors have nothing to report.

## Ethics Statement

This study was approved by the Ethics Committee of Alfonso X el Sabio University (Madrid, Spain) (Code: 2025_02/349).

## Consent

Informed consent was obtained from all participants.

## Conflicts of Interest

The authors declare no conflicts of interest.

## Data Availability

The data that support the findings of this study are available from the corresponding author upon reasonable request.
